# Ecthyma Gangrenosum: *Escherichia coli* or *Pseudomonas aeruginosa*?

**DOI:** 10.3389/fmicb.2017.00953

**Published:** 2017-05-30

**Authors:** Mohamed Abbas, Stéphane Emonet, Thilo Köhler, Gesuele Renzi, Christian van Delden, Jacques Schrenzel, Bernard Hirschel

**Affiliations:** ^1^Division of Infectious Diseases, Department of Medical Specialties, Geneva University HospitalsGeneva, Switzerland; ^2^Infection Control Programme, Geneva University HospitalsGeneva, Switzerland; ^3^Bacteriology Laboratory, Division of Infectious Diseases, Geneva University HospitalsGeneva, Switzerland; ^4^Department of Microbiology and Molecular Medicine, University of GenevaGeneva, Switzerland

**Keywords:** ecthyma gangrenosum, *Pseudomonas aeruginosa*, molecular diagnosis of infectious diseases, blood cultures, septic shock

## Abstract

**Background:** Ecthyma gangrenosum (EG) are necrotic lesions that develop in the context of *Pseudomonas aeruginosa* bacteremia. Isolated reports describe EG in the setting of non-Pseudomonal infections. In a patient with EG, initial blood cultures showed *Escherichia coli*, and almost occulted *P. aeruginosa* bacteremia. Based on the clinical picture we suspected preponderant *P. aeruginosa* bacteremia, outgrown by concomitant low-grade *E. coli* bacteremia in the blood culture vials.

**Methods**: We performed quantitative polymerase chain reaction (PCR) assays with specific primers for *P. aeruginosa* and *E. coli* on blood collected at the same time for blood cultures. We also performed quantitative cultures of the strains isolated from the patient’s blood.

**Results**: Quantitative PCR showed that there were 1.5 × 10^E^7 copies/milliliter (ml) of *P. aeruginosa* DNA, whereas the quantity of *E. coli* DNA was below the detection limit of 2 × 10^E^4 copies/ml. We estimated that there was at least 1000 times more *P. aeruginosa* than *E. coli*. Quantitative cultures showed that *E. coli* grew faster than *P. aeruginosa*.

**Conclusion**: Our patient with EG had preponderant *P. aeruginosa* bacteremia, that was almost occulted by concomitant low-grade *E. coli* bacteremia. Quantitative PCR was complementary to blood cultures in the final microbiological diagnosis, and proved beneficial in establishing the etiology of EG. This may question the existence of non-Pseudomonal EG, and also shows that blood culture results do not always reflect an “exact picture” of what happens in the patient’s blood at the time of sampling. This case illustrates the importance of communication between the clinician and the microbiology laboratory to ensure best possible results.

## Introduction

A 53-year-old man, whose medical history is significant for rheumatoid arthritis treated with corticosteroids, followed by methotrexate and adalimumab. He was diagnosed 4 months before his current hospitalization with glioblastoma after experiencing a seizure. He was treated by radiotherapy and temozolomide, and was hospitalized 1 month later for asthenia and thrombocytopenia, leading to discontinuation of temozolomide. Brain MRI showed tumor progression, for which pulses of methylprednisolone were given, followed by high doses of dexamethasone.

**FIGURE 1:**
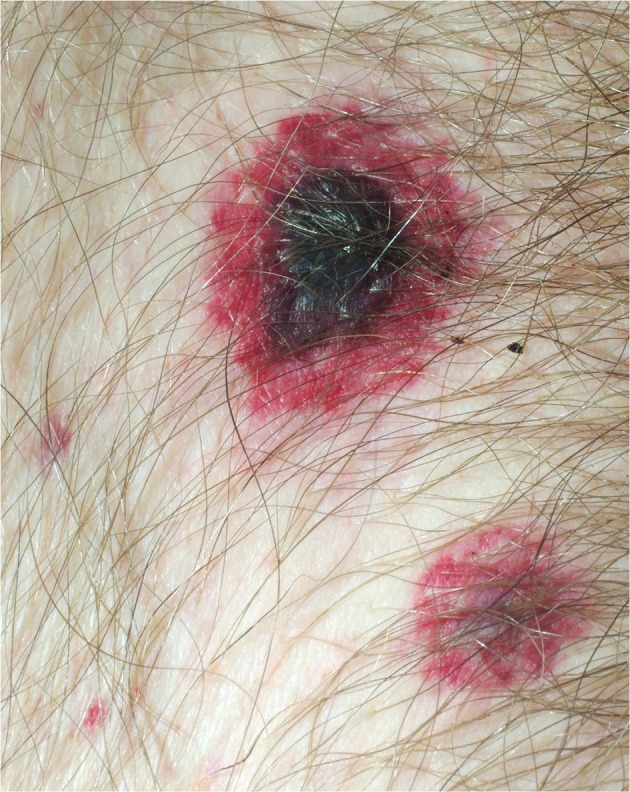
Photograph of a suprapubic ecthyma gangrenosum lesion. Notice the necrotic center of the large lesion, with an erythematous halo. The smaller lesion is presumed to be of an earlier stage.

On day 4 of hospitalization, the patient developed neutropenia, for which Granulocyte Colony Stimulating Factor (G-CSF) was administered. On day 7, he developed neutropenic sepsis for which imipenem/cilastatin was started. On the same day, several skin lesions appeared in the suprapubic area, the right leg, the left popliteal region, and the back (**Figure 1**). These were purpuric erythematous nodules with a necrotic center, and from 1 to 7 cm in diameter. The clinical suspicion was EG due to *Pseudomonas aeruginosa* bacteremia, therefore amikacin was added, but the patient’s condition rapidly deteriorated, and he died on day 12.

Biopsies of the skin lesions were also obtained, showing necrosis of dermis, hypodermis, and basal epidermis, with massive extravasation of red blood cells, and necrotic and thrombosed blood vessels. Vessels situated outside of the necrosis did not show signs of vasculitis. Separation of the epidermis from the dermis was present. Cultures were positive for *P. aeruginosa.*

The blood cultures revealed Gram-negative rods after 24 h, identified as *E. coli* the following day. Retrospective-search for *Pseudomonas* in the initial blood cultures allowed identification of one lactose negative colony on the MacConkey agar, which proved to be *P. aeruginosa* by Matrix Assisted Laser Desorption/Ionization Time-of-Flight Mass Spectrometry (MALDI-TOF MS, Microflex^TM^, Bruker Daltonics, Germany). It was hypothesized that there was a preponderant *P. aeruginosa* bacteremia, causing the EG, but that concomitant low grade *E. coli* bacteremia had outgrown *P. aeruginosa* in the blood culture vials.

To provide evidence for this hypothesis, we performed molecular biology assays and quantitative cultures. Written informed consent to publish this case report anonymously, without identifying information, was provided by the deceased patient’s spouse.

### Methods

#### Genomic DNA Extraction from Whole Blood (EDTA Tube)

We obtained a whole blood sample drawn on the same day as the first blood cultures, from which we extracted genomic DNA from four independent 100 microliter (μl) aliquots using a DNeasy^®^ Blood and Tissue Kit according to the manufacturer’s instructions (Qiagen, Hildesheim, Germany).

#### Quantitative Polymerase Chain Reaction (qPCR) Assay

We determined the number of *P. aeruginosa* and *E. coli* genomic copies in the patient’s blood by qPCR performed on the genomic DNA preparations, using primers specific for *P. aeruginosa* (rpsL-F: 5′-GCAAGCGCATGGTCGACAAGA-3′/rpsL-R: 5′-CGCTGTGCTCTTGCAGGTTGTGA-3′) (Dumas et al., 2006) and *E. coli* (UAL1939b: 5′-ATGGAATTTCGCCGATTTTGC-3′/UAL2105b: 5′-ATTGTTTGCCTCCCTGCTGC-3′) (Maheux et al., 2009). The reaction contained 3 μl of genomic DNA, primers at 600 nM each and 1x of Quantitect Sybr Green Master Mix (Qiagen) in a total volume of 15 μL. Duplicate samples were run in a RotorGene 3000 (Corbett Research, Sydney, NSW, Australia) as described previously (Dumas et al., 2006). The amount of genomic DNA copies in the blood samples was calculated by comparison with standard curves of *P. aeruginosa* and *E. coli* genomic DNA preparations. The specificity of the primers was verified on the *P. aeruginosa* and *E. coli* genomic DNA preparations (as *P. aeruginosa* culture samples were not amplified using the UAL1939b and UAL2105b (*E. coli*) primer pair, and *E. coli* culture samples were not amplified using the *rpsL* F/R (*P. aeruginosa*) primer pair).

#### Quantitative Culture Methods

We thawed *E. coli* and *P. aeruginosa* isolates from our patient (A), as well as isolates from unrelated patients (B + C) to make sure that we were not confronted to strain-specific behavior. Identification was confirmed by MALDI-TOF MS. A loopful of bacteria was inoculated in sterile NaCl 0.9% and diluted successively using McFarland turbidity. To allow quantification of the inoculum, each dilution was streaked on sheep blood agar at the time blood culture vials were inoculated. We seeded 8 milliliters (ml) of *E. coli* and of *P. aeruginosa* suspension from each patient (A, B, and C) in six different aerobic blood culture vials. We added 4 ml of *E. coli* (100 cfu/ml) and 4 ml of *P. aeruginosa* (1000 cfu/ml) from each patient (A, B, and C) to a blood culture vial (total of three vials), placed them into a BACTEC blood culture system (Becton-Dickinson), and recorded time to positivity (TTP). At that point, 100 μl of each culture dilutions were spread on MacConkey and blood agars. *E. coli* (pink colonies) and *P. aeruginosa* (colorless colonies) were quantified by CFU counts after 16 h of incubation at 37°C.

### Results

The standard curves for *P. aeruginosa* using the *rpsL* F/R primer pair showed a linear correlation between the amount of bacteria determined on the genomic DNA extracts and the number of cfu added. Identical results were observed for *E. coli* using the UAL1939b and UAL2105b primer pair. The four genomic DNA preparations from the patient’s blood yielded highly reproducible curves when amplified with the *P. aeruginosa* primer pair, with similar threshold values. The number of genomic copies of *P. aeruginosa* was 1.5 (±0.045)^∗^10^7^/ml. We detected no genomic copies of *E. coli* in sample A after amplification of the genomic DNA preparations using the *E. coli* primer pair, with a detection threshold of 2^∗^10^4^ copies/ml blood. Based on this detection level, we estimated that in the patient’s blood, the concentration of *P. aeruginosa* DNA was at least 1000 times more elevated than the concentration of *E. coli* DNA.

The quantitative cultures showed that the TTP for *E. coli* was 9–10 h, as compared to 15–16 h for *P. aeruginosa*. After mixed seeding, using MacConkey agar, isolated *P. aeruginosa* colonies were buried in a mass of *E. coli* colonies, and could easily have been “missed” if the laboratory technician wasn’t specifically alerted to the possibility of their presence. Therefore, despite an initial inoculum of 4000 cfu/ml of *P. aeruginosa* compared to 400 cfu/ml of *E. coli*, the latter outcompeted *Pseudomonas* in the mixed blood culture vial as well as on the MacConkey agar.

## Background

Ecthyma gangrenosum (EG) was the name given to skin lesions that “evolved from erythematous macules or vesicles into necrotic ulcers” associated with *Bacillus pyocyaneus* (former name of *P. aeruginosa*) (Greene et al., 1984). Most reports of EG involve patients with disorders of the innate immune system, such as hematological malignancies or drug-induced neutropenia (Dorff et al., 1971; Greene et al., 1984; Reich et al., 2004). While EG is pathognomonic of *P. aeruginosa* bacteremia, reports of EG or EG-like lesions associated with other microorganisms have recently been extensively reviewed and summarized (Vaiman et al., 2015), and the authors suggest that broad definitions of EG should be adopted. It remains controversial whether EG is exclusively associated with *P. aeruginosa* or not. Our objective was to shed new light on this debate, and to illustrate the importance of communication between the clinician and the microbiology laboratory.

## Discussion

Despite initial blood cultures suggesting mono-bacterial *E. coli* bacteremia, we confirmed our suspicion of preponderant *P. aeruginosa* bacteremia by demonstrating that the quantity of *P. aeruginosa* DNA was at least 1000 times that of *E. coli*. Experimental quantitative cultures showed that the growth of *P. aeruginosa* was slower than that of *E. coli* when cultivated separately, and was completely overwhelmed by the growth of *E. coli* when cultivated together. It has previously been reported that *P. aeruginosa* has longer doubling times in the presence of other bacteria, perhaps due to competition for nutrients (Tamagnini and González, 1997).

These results suggest that blood cultures might sometimes yield a biased vision of what is in the patient’s blood. Reports of the clinical application of molecular diagnosis of sepsis suggest that the combination of PCR and blood cultures is superior to either technique alone, and may thus be complementary (Lamoth et al., 2010; Lehmann et al., 2010). Real-time PCR identified microorganisms that were unidentified by culture, particularly in patients who were receiving antibiotics at the time of sampling (Lamoth et al., 2010; Pasqualini et al., 2012). Molecular diagnosis of sepsis has limitations that make its implementation in routine diagnostic laboratory methods impossible at the moment (detection thresholds, interpretation of DNAemia) (Emonet and Schrenzel, 2011). Newer methods of molecular diagnosis are being evaluated, such as PCR followed by electrospray ionization-mass spectrometry (PCR/ESI-MS), with promising prospects, yet they also require further evaluation (Vincent et al., 2015).

It is controversial whether ecthyma-like lesions due to pathogens other than *P. aeruginosa* should be called EG. Some believe that EG is “specifically associated with systemic *P. aeruginosa* infection” (Dorff et al., 1971; Greene et al., 1984). For example, Hurwitz states that “it may be better to avoid the name EG for these localized, progressive ulcerative infections because they may not be the result of a bacteremia or septicemia, and they may not be due to a *Pseudomonas* organism” (Hurwitz, 1987). We believe it is legitimate to ask whether the reported cases of non-Pseudomonal EG cases represent mixed bacteremia with undetected *P. aeruginosa*, and therefore adhere to the original definition (Dorff et al., 1971; Greene et al., 1984; Hurwitz, 1987).

## Concluding Remarks

This case teaches us that even results from a “gold standard” test may be equivocal, and highlights the added value of a clinical diagnosis to a microbiology laboratory’s routine work; regular communication between laboratory and clinician is a win-win situation, which ultimately benefits the patient (Baron et al., 2013).

## Author Contributions

All the authors provided substantial contributions to the conception and design of the work. MA and TK designed, performed and interpreted the results the quantitative PCR experiments under the supervision of CvD. SE and GR designed, performed and interpreted the results of the quantitative culture experiments under the supervision of BH. All authors participated actively in the interpretation of the microbiological results, as well as the drafting of the manuscript. All authors approved the final version of the manuscript, and agree to be accountable for all aspects of the work in ensuring that questions related to the accuracy or the integrity of any part of the work are appropriately investigated and resolved.

## Conflict of Interest Statement

The authors declare that the research was conducted in the absence of any commercial or financial relationships that could be construed as a potential conflict of interest.
